# High-resolution mapping of chromatin conformation in HBeAg-treated macrophage provides insights into pathogenesis of HBV-related liver diseases

**DOI:** 10.3389/fimmu.2026.1871642

**Published:** 2026-07-16

**Authors:** Tiantian Liu, Xiaoyu Xie, Shujun Ma, Huiling Cao, Wenwen Wang, Zhen Yu, Yuemin Feng, Jianni Qi, Hongjun Bian

**Affiliations:** 1 Shandong Provincial Hospital Affiliated to Shandong First Medical University, Jinan, Shandong, China; 2 Provincial Hospital Affiliated to Fuzhou University, Fuzhou, Fujian, China; 3 Department of Gastroenterology, Qilu Hospital of Shandong University, Jinan, Shandong, China; 4 University Town Hospital, Affiliated Hospital of Shandong University of Traditional Chinese Medicine, Jinan, Shandong, China; 5 Shandong Provincial Engineering and Technological Research Center for Liver Diseases Prevention and Control, Jinan, Shandong, China

**Keywords:** HBeAg, Hi-C, liver, macrophage, three-dimensional (3D) chromatin architecture

## Abstract

**Background:**

Hepatitis B virus (HBV) e antigen (HBeAg) plays a critical role in inducing macrophage activation and subsequent immune tolerance, which facilitates viral immune escape. However, the underlying spatial 3D genomic and epigenetic mechanisms driving this macrophage dysfunction remain largely unknown.

**Methods:**

To map the topological and transcriptional regulatory landscape, we integrated RNA-sequencing (RNA-seq), high-throughput chromosome conformation capture (Hi-C), and chromatin immunoprecipitation-sequencing (ChIP-seq) to comprehensively analyze control and HBeAg-stimulated macrophages. Key findings were cross-validated using human HBV-infected datasets.

**Result:**

HBeAg robustly activated pro-inflammatory transcriptional programs, prominently featuring the TNF signaling pathway as a central node. Hi-C analysis revealed profound global 3D chromatin reorganization accompanying this phenotypic shift. Specifically, inactive-to-active (B-to-A) compartment switching, coupled with *de novo* H3K27ac enhancer accumulation, directly drove the upregulation of functional genes such as MET and FHOD3. Furthermore, topologically associating domain (TAD) restructuring, particularly TAD merging, exposed new regulatory elements to upregulate FLNB and SESN2. Conversely, the disruption of specific intrachromosomal loops resulted in the targeted downregulation of genes like KBTBD11 and BLVRB. Mechanistically, targeted epigenetic reprogramming drove this 3D structural rewiring: H3K27ac enhancer deposition was enriched for AP-1/STAT motifs, CTCF coordinated with FOX-family factors to alter structural boundaries, and HLTF mediated targeted H3K27me3-associated gene silencing.

**Conclusion:**

HBeAg orchestrates a highly coordinated hierarchical restructuring of the 3D genome and targeted epigenetic reprogramming to induce macrophage dysfunction. The identified structural variants and core spatial-target genes (such as MET, FLNB, and BLVRB) provide novel mechanistic insights and represent potential therapeutic targets for HBV-related liver diseases.

## 1 Introduction

Hepatitis B is a public social problem that seriously threatens the health of people around the world. About 2 billion people are infected hepatitis B viruses (HBV) and more than 350 million are chronic carriers of HBV (1, 2). After HBV infection, it will induce persistent inflammatory response in the liver and repeated death and proliferation of liver cells, eventually leading to liver cirrhosis and hepatocellular carcinoma (2). Serum hepatitis B e antigen (HBeAg) levels are associated with viral replication, infectivity, inflammation, disease severity, and response to antiviral therapy. Our previous work demonstrated that compared with hepatitis B c antigen (HBcAg) and HBsAg, HBeAg is the most important element in HBV-associated antigens that induce macrophage activation, and it also promotes activation of stellate cells through cell-cell crosstalk, ultimately affecting the progression of the liver disease (3–7).

A complex chromatin regulatory network affects the 3D chromatin structure of the human genome and plays an important role in transcriptional changes during human development. Differentiation of monocytes into mature macrophages is regulated by several key transcriptional regulators, including SPI1, MAFB, and AP-1 (8, 9). At the same time, Kevin Van Bortle et al. demonstrated that 3D chromatin structural changes during the differentiation of human monocytes into macrophages by analyzing comprehensive *in situ* chromosome conformation capture (Hi-C) maps of DNA loops. The differentiation and maturation of human monocytes is accompanied by changes in DNA loops associated with key regulatory genes important for macrophage development and function (10). In addition, the study has also shown that the genome structure undergoes a large-scale remodeling during the transformation of primary human monocytes to macrophages, in which about 90% of topologically associated domains (TADs) are changed. These TAD changes correlated with changes in immune gene expression (11). However, the chromosomal conformational changes during HBeAg-treated macrophage activation are not well understood. By understanding the changes of 3D genome organization in HBeAg-treated macrophage, it will help us to better understand the pathogenesis of various liver diseases caused by HBV infection, and then provide a theoretical basis for clinical treatment research.

There are two main technologies for exploring the spatial structure of 3D genomes, including DNA fluorescence *in situ* hybridization (DNA-FISH) based on imaging technology and high-throughput chromosome conformation capture (Hi-C) technology based on chromosome conformation capture (3C) (12). The three-dimensional organization of the genome has emerged in recent years as an important player in gene expression regulation. Hi-C sequencing technology is a 3C-based method, which obtains 3D genome information of a disease by detecting changes in chromatin interactions and hierarchical structures, like alterations of A/B compartment, TAD boundary, and chromatin loop (13). *In situ* Hi-C enables higher resolution at currently achievable sequencing depths. However, there is still a lack of relevant research on the dynamic changes in the activation and differentiation of macrophages caused by HBeAg treatment.

In this study, we utilized the well-established murine RAW264.7 macrophage line to perform a comprehensive analysis of HBeAg-induced 3D genomic changes, combining Hi-C, ChIP-seq, and RNA-seq data. While previous studies have examined HBV infection broadly, we focused specifically on HBeAg due to our previous findings identifying it as the primary driver of macrophage activation and cytokine release compared to HBsAg and HBcAg (3–5). Furthermore, by cross-validating our murine data with human THP-1 and HBV patient datasets (GSE179618, GSE83148), we aimed to identify evolutionarily conserved, 3D genome-driven transcriptional programs. These findings demonstrate a complex interplay between epigenetic landscapes and spatial chromatin architecture, proposing new mechanistic hypotheses regarding how HBeAg drives macrophage-mediated liver inflammation.

## 2 Methods

### 2.1 Cell culture and reagents

Mouse macrophage cell line RAW264.7 was obtained from the American Type Culture Collection (ATCC) and cultured in DMEM supplemented with 10% FBS at 37 °C with 5% CO2. Recombinant HBeAg (ab91273, Abcam, Cambridge, UK) was reconstituted according to the manufacturer’s protocol. To avoid confounding effects from non-specific bacterial endotoxins, the HBeAg preparation was verified to contain <0.1 EU/μg of endotoxin utilizing a Limulus Amebocyte Lysate (LAL) assay, and Polymyxin B (10 μg/mL) was added to all cultures to neutralize any trace endotoxins. RAW264.7 macrophages were stimulated with HBeAg at a working concentration of 1 μg/mL for 24 h (Treatment group, T). The control group (Control group, C) received an equivalent volume of vehicle buffer (PBS) in parallel for 24 h.

### 2.2 RNA-seq and data analysis

Two independent biological replicates of control and HBeAg-treated macrophages were collected into TRIZOL for RNA extraction. While classical differential expression necessitates a larger sample size, robust estimation of dispersion was achieved using the DESeq2 package, which pools information across genes to handle small replicate numbers. Clean reads were mapped to the mouse reference GRCm38 genome using HISAT2. Differential gene expression was analyzed using DESeq2, with significant genes defined by an absolute |log2 Fold change| ≥ 1 and an adjusted P-value (FDR) < 0.05.

### 2.3 GEO datasets and Hi-C data processing

Transcriptomic data of GSE179618 (THP-1 cells) and GSE83148 (liver tissue) were obtained from the GEO database. To allow for accurate comparison across species and platforms, raw count matrices from these datasets were processed through the same normalization pipeline (DESeq2) as our internal data. Hi-C libraries (n=2 biological replicates) were prepared according to the protocol provided by Rao et al. with slight modifications (14). Cells were cross-linked by 1% formaldehyde and then lysed using lysis buffer (10 mM Tris-HCl pH 8.0, 10 mM NaCl, 0.2% Igepal CA-630, 1/10 volume protease inhibitor cocktail (Sigma)). Chromatin digestion was then performed using restriction enzymes (HindIII/MboI). After biotin labeling, blunt end ligation and DNA purification and extraction, Hi-C samples were prepared. The Hi-C fragment is obtained by removing the end-labeled biotin, ultrasonic interruption, end repair, adding base A, removing the fragment containing biotin, adding a sequencing adapter to form the adapter product, and then screening the Hi-C fragment to amplify the PCR conditions to obtain the HIC library. After the constructed library passed the library quality control, it was sequenced with Illumina HiSeq using the PE150 detection strategy. By removing sequences containing sequencing adapters and low-quality sequences from the original off-machine data, filtering to obtain high-quality Clean Reads, and then performing subsequent analysis, all subsequent analyses are based on Clean Reads. We used HiC-Pro to process the raw data to analyze the mapped reads and obtain the corresponding normalized interaction matrix, while identifying valid and invalid interaction pairs. Furthermore, the normalization of the matrix is achieved by an iterative correction method.

### 2.4 A/B compartment analysis and TAD analysis

We identified compartments A/B using normalized interaction matrices at 1MB resolution by using cworld-dekker software obtained from GitHub (https://github.com/dekkerlab/cworld-dekker) matrix2compartment.pl script. The chromatin region was divided into two spatially separated compartments according to the first principal component. Among them, the A compartment is the compartment with higher gene density and positive principal component analysis (PCA) value, and the B compartment is the compartment with lower gene density and negative PCA value. Additionally, matrix2insulation.pl in the cworld-dekker software was used to identify TAD boundaries at 20 kb resolution.

### 2.5 ChIP-seq and data analysis

ChIP experiments (n=2 biological replicates per condition) were performed according to the protocols of the EZ-Magna ChIP™ A kit (Millipore#17-408). Then, control samples and HBeAg-treated samples were immunoprecipitated with 10ug of the corresponding antibodies (CTCF Millipore #07-729; H3K27me3 Millipore #17-622; H3K27ac Millipore #17-683; p300 Millipore #05-257). DNA purified from CTCF, H3K27me3, H3K27Ac, and p300 ChIP assays was adapter-ligated and PCR amplified for sequencing on the Illumina HiSeq X Ten sequencer. After sequencing, data were aligned to mouse GRCm38 genome by Bowtie version 2.1.0 (http://bowtie-bio.sourceforge.net/bowtie2/index.shtml) and peak calling was performed using MACS2 (version 2.1.1). At the same time, peak annotation is performed by bedtools ver 2.20.1 (http://bedtools.readthedocs.io/en/latest).

### 2.6 Motif analysis

We utilized MEME to detect the most enriched significant motif sequences. Specifically, sequence characteristics natively differ between transcription factors and epigenetic marks; therefore, motif analysis was exclusively performed on the top 1000 dynamically changed peaks identified in the CTCF and H3K27ac datasets, as these are primary drivers of chromatin looping and enhancer activation, respectively. Enrichment was sorted by adjusted p-values.

### 2.7 Statistics

Advanced volcano plot, advanced Heatmap barplot and enrichment Scatter Plot were performed using the OmicStudio tools at https://www.omicstudio.cn/tool. Gene interaction network was drawn using cytoscape v3.7.2. Other statistical graphs were drawn using Graphpad prism 8.0.2. We performed data analysis using the Mann-Whitney test (unpaired) or the Wilcoxon paired signed-rank test (paired). Unpaired multiple comparisons were analyzed using the Kruskal-Wallis test followed by Tukey’s test, and paired multiple comparisons were analyzed using the Friedman test followed by Dunn’s test (paired). All analyses were performed using R 4.0. P<0.05 was considered statistically significant.

## 3 Result

### 3.1 HBeAg robustly activates inflammatory transcription programs in macrophages

**Figure 1:**
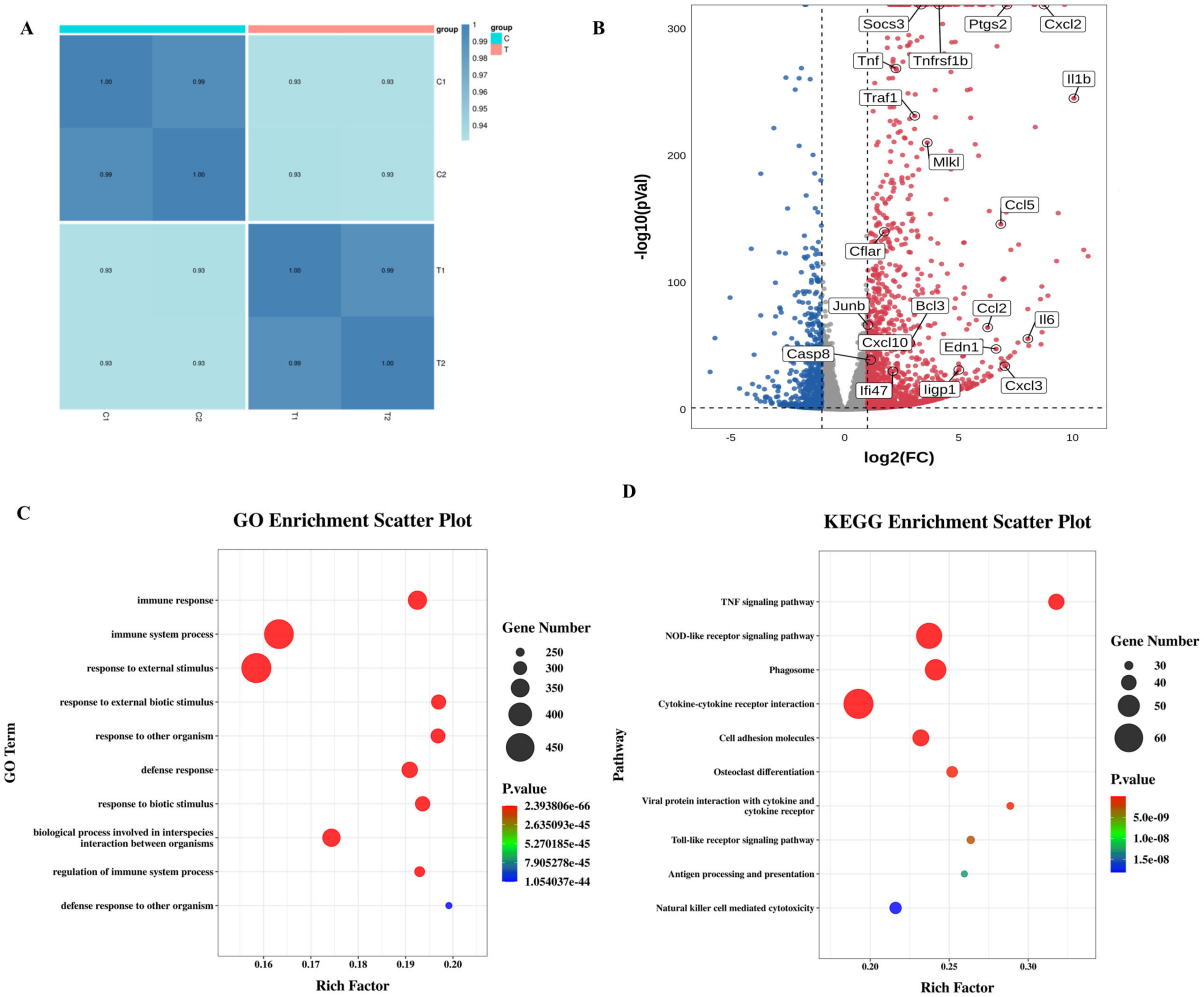
HBeAg induces a pro-inflammatory transcriptional program in macrophages. **(A)** Gene expression correlation heat map. **(B)** Differential gene volcano plot. **(C)** Top 10 GO enriched pathways. **(D)** Top 10 KEGG enriched pathways.

To map the transcriptional rewiring induced by HBeAg in macrophages, RNA-seq was performed. Following stringent quality control (**Supplementary Table 1**), PCA and correlation heatmaps demonstrated high reproducibility between biological replicates despite the sample size (n=2) (**Figure 1A**). We identified 2, 890 differentially expressed genes (FDR < 0.05), of which 2, 035 were upregulated (**Figure 1B**). Meanwhile, the volcano plot showed that inflammation-related genes were significantly upregulated in HBeAg-stimulated cells (**Figure 1B**). Gene Ontology and KEGG pathway analysis highlighted a profound shift toward an inflammatory phenotype, characterized by activation of the TNF signaling pathway, cytokine-receptor interactions, and Toll-like receptor signaling (**Figures 1C, D**). This is consistent with our previous research results (4, 7). Notably, TNF emerged as a central node in the dysregulated gene network (**Supplementary Figure 1A**). This may be related to the fact that TNF can promote immune tolerance through various mechanisms, including exacerbating T cell exhaustion and stimulating regulatory T cells to produce immunosuppressive factors (15). Since the main reason for the poor efficacy of current antiviral treatment for hepatitis B is that HBV escapes innate immunity, the role of TNF in inducing immune tolerance may be a possible mechanism for HBV immune escape (16). In addition, we performed a gene expression pattern clustering diagram on the differential genes screened out by gene expression difference analysis (**Supplementary Figure 1B**). The results showed that the gene expression differences between cluster 3 and cluster 6 were significant, and the specific gene lists were supplemented in **Table 2**. Collectively, these data indicate that HBeAg effectively induces a specific pro-inflammatory transcriptional program in macrophages.

### 3.2 HBeAg induces global 3D chromatin reorganization

**Figure 2:**
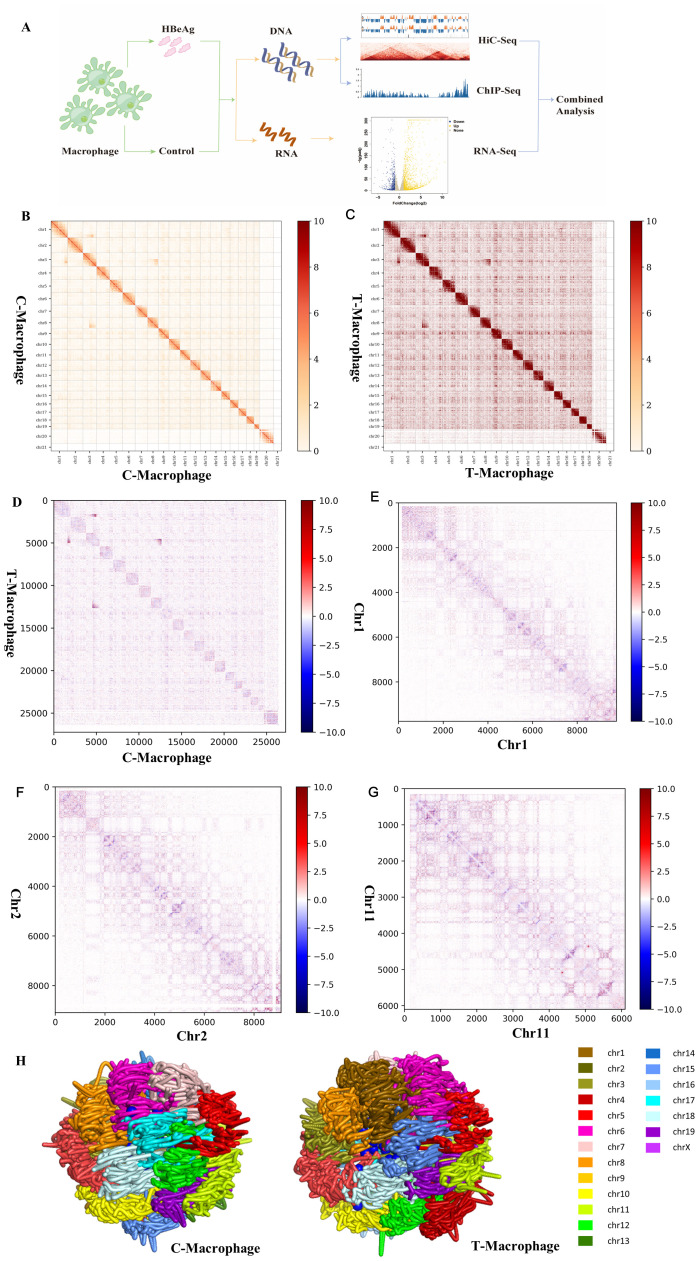
HBeAg triggers global three-dimensional chromatin reorganization. **(A)** Schematic representation of the experimental strategy. **(B, C)** Heatmaps of genome-wide interaction matrices of control macrophages and HBeAg-stimulated macrophages. **(D)** Interaction subtraction matrices of the whole genome between the two groups of samples. **(E–G)** Interaction subtraction matrices of single chromosomes between the two groups of samples (chr1, chr2, and chr11). **(H)** Three-dimensional model of chromosome interactions between control macrophages and HBeAg-stimulated macrophages.

Macrophages from the HBeAg-treated and control groups were cross-linked to map the 3D chromatin conformation (**Figure 2A**). More than 486 million clean double-end reads were obtained for each sample, with valid pairs accounting for 71.06% and 57.51% of clean reads in the control (C) group and 67.61% and 67.56% of clean reads in the HBe-Ag-treated (T) group (**Supplementary Table 3**). In Hi-C analysis, the minimum value of the bin to which Hi-C data can be assigned is the resolution, and the resolution is related to the endonuclease used in the Hi-C experiment and the amount of sequencing data. The reference genome is divided based on the size of the bin, and the valid pairs are assigned to the bin to construct the original interaction matrix. A 100-kb resolution whole-genome interaction matrix was constructed for each sample and corrected. The corrected whole-genome interaction matrix was displayed in the form of a heat map (**Figures 2B, C**). In addition, in order to more intuitively compare and display the interaction differences between samples, we show the interaction subtraction matrix of the whole genome at 100kb resolution between the two groups of samples (**Figure 2D**). To intuitively visualize these global structural alterations, we plotted the interaction subtraction matrix. Chromosomes 1, 2, and 11 were selected for high-resolution (20kb) 2D modeling because they exhibited the highest density of differential structural variance following HBeAg treatment, serving as representative models of genome-wide reorganization (**Figures 2E–G**). In addition, we also drew a three-dimensional model of chromosome interactions (**Figure 2H**; **Supplementary Videos 1, 2**). These global interaction changes strongly suggest that HBeAg-induced macrophage activation is accompanied by profound 3D chromatin reorganization.

### 3.3 A/B compartment switching correlates with enhancer activation and up-regulation of Met and Fhod3

The high-resolution resources generated by Hi-C can also explore other aspects of changes in the physical environment at genomic loci in HBeAg-treated macrophages, such as A/B compartments, TAD, and chromatin cycling. Lieberman-Aiden found that the mammalian genome can be divided into two compartments, called A/B compartments, in which the A compartment is more closely related to open chromatin, and the B compartment is more closely related to closed chromatin (17). In addition, A/B compartments are cell-specific and can switch in different tissues and cells, and this switch has a certain relationship with gene expression regulation.

**Figure 3:**
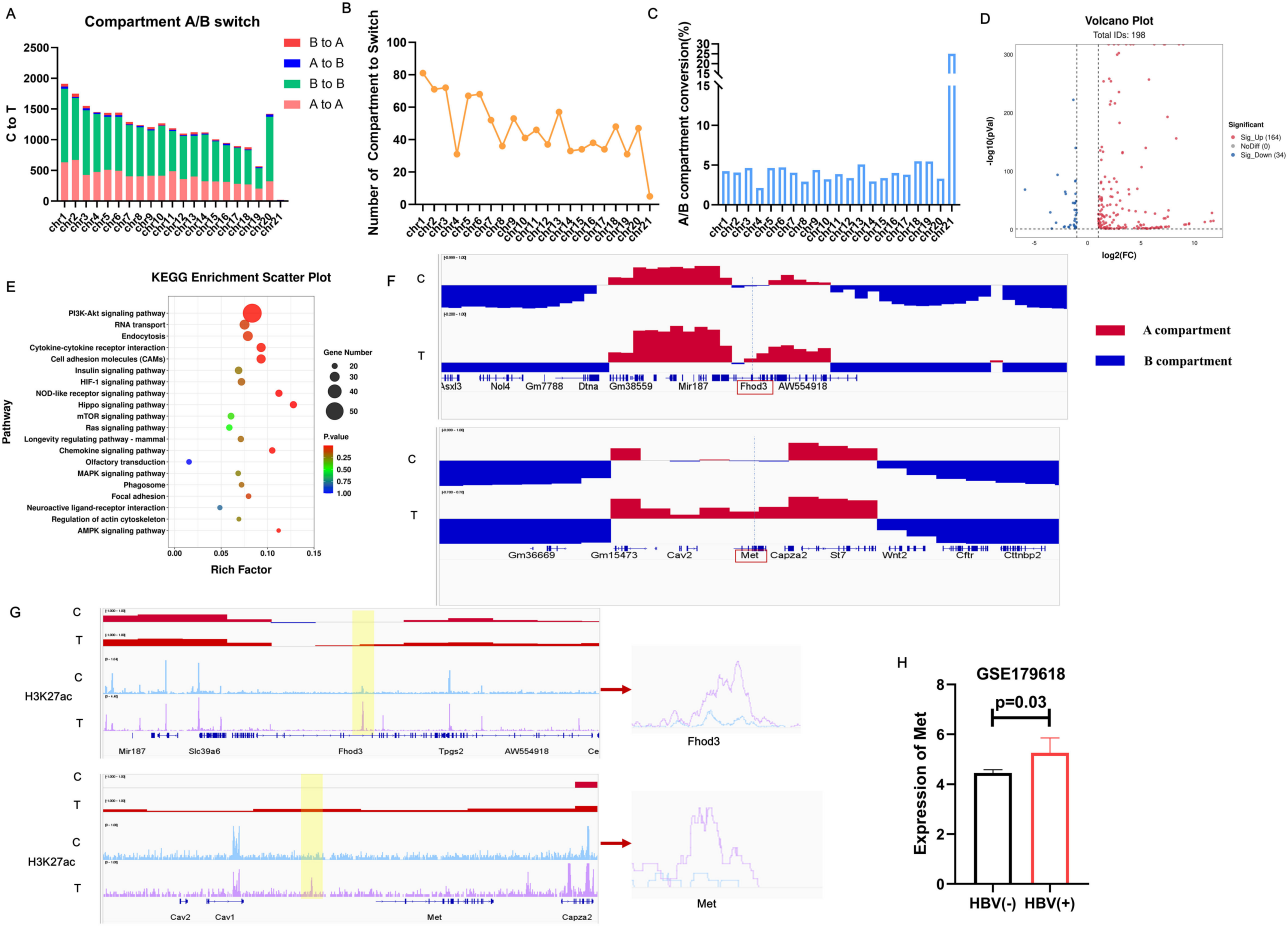
A/B compartment switching coupled with enhancer activation drives the targeted upregulation of specific genes. **(A–C)** Summary of the conversion of A/B compartments on each chromosome in macrophages of the HBeAg stimulation group. **(D)** Volcano plot of differentially expressed genes related to A/B compartment conversion. **(E)** Top 20 KEGG enriched pathways related to differentially expressed genes related to A/B compartment conversion. **(F)** Examples of differentially expressed genes related to A/B compartment conversion in macrophages of the HBeAg stimulation group (FHOD3, MET). **(G)** Examples of differentially expressed gene regions where H3K27ac signals are significantly enriched in A/B compartment switching (FHOD3, MET). **(H)** MET expression was analyzed based on the data of GSE179618. C: macrophages of the control group; T: macrophages of the HBeAg stimulation group.

To understand differences in chromatin interactions in HBeAg-treated macrophage genomes, we separated chromatin into A/B compartments by principal component analysis (PCA). We compared and summarized the conversion of A/B compartments on each chromosome in macrophages of the control group and HBeAg-stimulated group (**Figures 3A–C**; **Supplementary Table 4**). The chromosomes with the most A/B compartment conversion in macrophages of the HBeAg-stimulated group were chr1, chr2, chr3, chr5 and chr6 (**Figure 3B**), and the chromosomes with the most A/B compartment conversion ratio were chr6, chr13, chr18, chr19 and chr21 (**Figure 3C**). Combined with RNA-seq data, the relationship between different compartments and gene expression was analyzed to show the differential genes caused by compartment conversion (**Supplementary Table 5**). Unifying Hi-C architecture with transcription, we identified 198 genes whose expression directly mirrored compartment transitions, including 164 up-regulated genes and 34 down-regulated genes (**Figure 3D**). At the same time, these differentially expressed genes were enriched in multiple inflammation-related and macrophage function-related pathways, including the PI3K-Akt pathway, endocytosis, and cytokine-cytokine receptor interaction (**Figure 3E**).

Crucially, combining this with our ChIP-seq data, we observed that regions shifting from inactive (B) to active (A) compartments were accompanied by *de novo* accumulation of H3K27ac—an active enhancer mark.For example, Met and Fhod3 transitioned from B to A compartments and exhibited robust increases in H3K27ac enhancer signals, resulting in their significant transcriptional upregulation (**Figures 3F, G**). To validate the translational relevance of these findings, we queried human datasets and found that MET was similarly upregulated in HBV-stimulated human THP-1 macrophages (**Figure 3H**). This cross-species conservation strongly suggests that HBeAg-driven A/B compartment flipping acts as an epigenetic switch to activate specific gene programs. This indicates that A/B compartment switching, coupled with enhancer activation, precisely dictates target gene transcription.

### 3.4 Identification of alternations in TADs and related gene expression in HBeAg-treated macrophage

**Figure 4:**
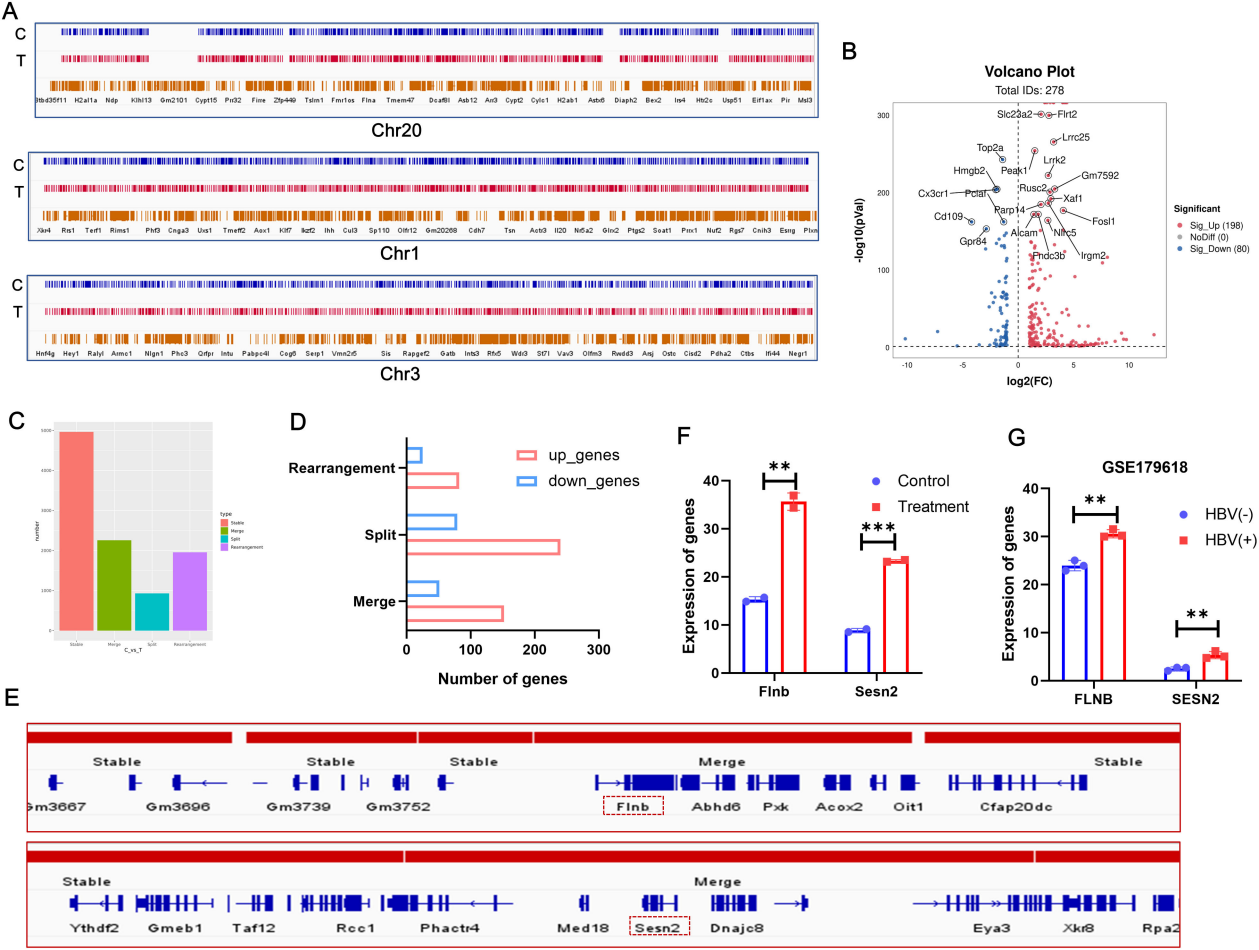
Topologically associating domain (TAD) restructuring by HBeAg alters the regulatory environment of target genes. **(A)** Display of TAD boundaries in control and HBeAg-treated macrophages (Chr1, Chr3, Chr20). **(B)** Volcano plot of differentially expressed genes associated with specific TAD boundaries in HBeAg-treated macrophages. **(C)** The number of four types of TADs (stable, merged, split, rearranged) in HBeAg-treated macrophages. **(D)** The number of differentially expressed genes associated with altered TADs in HBeAg-treated macrophages. **(E)** Examples of differentially expressed genes associated with TAD changes in macrophages in the HBeAg-stimulated group (SESN2, FLNB). **(F)** Expression levels of FLNB and SESN2 in control macrophages and HBeAg-treated macrophages. **(G)** Expression levels of FLNB and SESN2 in GSE83148. **p<0.01; ****p<0.001.

The genome is structured by splicing pieces of chromatin with strong local interactions, which are called “topological domains”. Interactions within TADs are strong, and interactions between different TADs are weak (17, 18). We identified TADs by insulation scoring at 20 kb resolution. We identified 10, 104 TADs and 9, 982 TADs in control macrophages and HBeAg-stimulated macrophages, respectively (**Supplementary Table 6**) and displayed the number of TADs per chromosome (**Supplementary Figure 2A**). There was no significant difference in TAD size between the two groups of macrophages. In addition, 10, 083 and 9, 962 TAD boundaries were detected in control macrophages and HBeAg-stimulated macrophages, respectively (**Supplementary Figure 2B**; **Supplementary Table 6**). The TAD boundaries that only exist in the macrophages of the stimulation group are called treatment group-specific boundaries (T-TADs), among which the number of T-TAD borders on chr20, chr1, and chr3 is the largest (**Supplementary Figure 2C**; **Figure 4A**; **Supplementary Table 7**).

The T-TAD border-related differentially expressed genes were displayed by joint analysis with RNA-seq (**Supplementary Table 8**), of which 198 genes were upregulated and 80 genes were downregulated (**Figure 4B**). In addition, compared with the treated samples, TADs in the control samples could be divided into four categories: Stable, Merge, Split, and Rearrangement (19). We identified 4, 964 Stable TADs, 2, 258 Merge TADs, 931 Split TADs, and 1, 957 Rearrangement TADs among all TADs (**Figure 4C**). At the same time, combined with RNA-seq analysis, we found that there were 152 up-regulated genes associated with Merge TAD and 79 down-regulated genes associated with Split TAD (**Figure 4D**; **Supplementary Tables 9, 10**). Notably, the filamin B (FLNB) and sestrin 2 (SESN2) loci were each encapsulated in a functional “merged TAD”, exposing them to novel regulatory elements, consistent with their significant upregulation of transcription (**Figures 4E, F**). Because immortalized murine cell lines have limitations, we validated this finding against human datasets. FLNB and SESN2 were significantly upregulated in human THP-1 cells treated with HBV (GSE179618) (**Figure 4G**), underscoring a conserved mechanism where HBeAg-induced TAD merging promotes genes associated with cytoskeletal remodeling and cell growth.

### 3.5 Chromatin loop alterations disrupt repressor loci and promote inflammatory signaling

**Figure 5:**
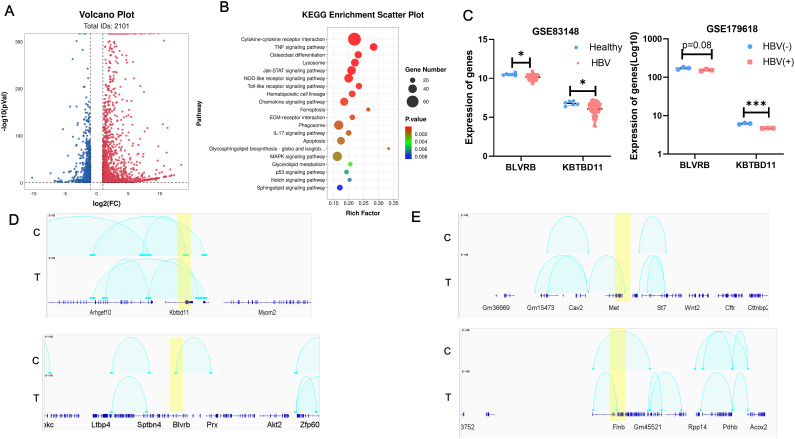
Alterations in chromatin looping disrupt repressive loci and facilitate the expression of core functional genes. **(A)** Volcano plot of differentially expressed genes associated with intrachromatin specific loops in HBeAg-treated macrophages. **(B)** Top 20 KEGG enriched pathways enriched for differentially expressed genes associated with intrachromatin specific loops in HBeAg-treated macrophages. **(C)** Gene expression levels of KBTBD11 and BLVRB in GSE83148 and GSE179618. **(D)** Examples of differentially expressed genes associated with chromatin loop changes in HBeAg-stimulated macrophages (KBTBD11 and BLVRB). **(E)** Changes in chromatin loops of core genes MET and FLNB associated with A/B compartment switching and TAD changes in HBeAg-stimulated macrophages. *p<0.05; ***p<0.001.

DNA sites that are linearly far apart on the genome sequence will be spatially close to each other to form loops, allowing different biological domains to approach each other for transcriptional regulation. We detected the loops structure on the chromatin and analyzed the changes in the loops structure on the chromatin of macrophages in the control group and HBeAg-treated macrophages. The numbers of intrachromosomal specific loops (C_cis) in macrophages of the control group and the HBeAg-treated macrophages (T_cis) were 68567 and 103499, respectively (**Supplementary Figure 2D**). The numbers of interchromosomal specific loops (C_trans) in macrophages of the control group and the HBeAg-treated macrophages (T_trans) were 44417 and 31211, respectively (**Supplementary Figure 2D**). In addition, we summarized the ratio of the number of intrachromosomal specific loops on each chromosome between HBeAg-treated and control samples (**Supplementary Figure 2E**; **Supplementary Table 11**). There were 2101 differentially expressed genes related to chromosomal loops specific to macrophages in the HBeAg-stimulated group, including 1496 up-regulated genes and 605 down-regulated genes (**Figure 5A**). KEGG enrichment analysis showed that these genes were mainly enriched in pathways such as Cytokine-cytokine receptor interaction, TNF signaling pathway, and Toll-like receptor signaling pathway, which was consistent with our previous study (**Figure 5B**).

Based on the unique loop-related differentially expressed genes in macrophages in the HBeAg-stimulated group, combined with GSE83148 and GSE179618 data analysis, it was found that kelch repeat and BTB domain containing 11 (KBTBD11) was downregulated in both liver tissues of HBV patients and HBV-treated THP-1 macrophage samples (**Figure 5C**). In addition, biliverdin reductase B (BLVRB) was downregulated in HBV-treated THP-1 macrophage samples and had a downward expression trend in HBV patients (**Figure 5C**). The expression of BLVRB in HBV patients showed a trend without statistical significance, which may be due to the small number of samples. At the same time, we also found that the loops in the KBTBD11 and BLVRB gene expression regions in macrophages in the HBeAg-stimulated group disappeared (**Figure 5D**). What’s more, the core genes MET and FLNB associated with A/B compartment switching and TAD changes were also found to undergo loop changes in HBeAg-stimulated macrophages (**Figure 5E**). In sum, these results suggest that changes in intrachromosomal interactions in HBeAg-stimulated macrophages may lead to changes in pathways related to macrophage function and inflammatory response. Changes in the expression of KBTBD11, BLVRB, MET, and FLNB involved in these pathways may be potential therapeutic targets for HBV-related liver disease.

### 3.6 HBeAg remodels the epigenetic landscape to facilitate enhancer-promoter communication

**Figure 6:**
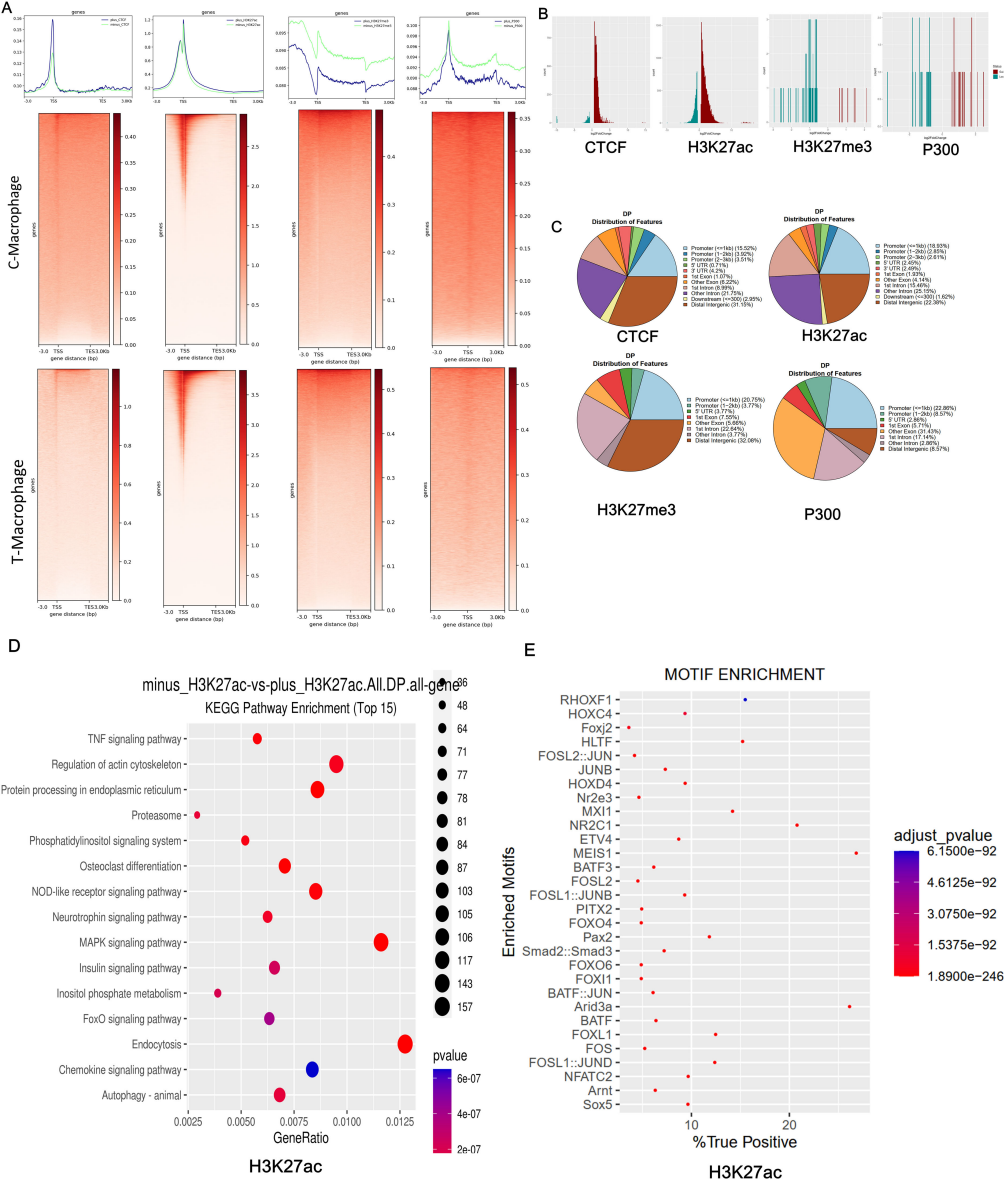
HBeAg drives targeted epigenetic reprogramming and specific enhancer activation. **(A)** Binding of CTCF, H3K27ac, H3K27me3 and P300 in the gene region from the transcription start site (TSS) to the transcription end site (TES). **(B, C)** Distribution of gain peaks and loss peaks in macrophages stimulated by HBeAg. **(D)** KEGG analysis of enriched pathways of genes associated with differential peaks H3K27ac. **(E)** Motif enrichment results related to gain peaks in H3K27ac.

Studies have shown that various protein factors play a role in chromatin folding required for normal gene expression (20). One such factor is CCCTC-binding factor (CTCF), a DNA-binding protein that induces chromatin recycling and binds at TAD boundaries (21). CTCF is integral to cell survival, and specific CTCF binding sites have been shown to influence gene expression in development, physiology, and cancer (22–25). We visually display the binding of CTCF, H3K27ac, H3K27me3 and P300 in the gene region from transcriptional start site (TSS) to transcriptional end site (TES) (**Figure 6A**). 9830 and 2220 peaks were detected in the control macrophage and HBeAg-treated macrophage, respectively (**Supplementary Table 12**). According to the peak detection results of each sample, the peaks were annotated and 2117 (CTCF), 16954 (H3K27ac), 27 (H3K27me3), and 22 (P300) genes were obtained in the macrophages of the HBeAg stimulation group, respectively (**Supplementary Table 12**). The numbers of specific peaks in macrophages of the HBeAg-stimulated group were 724 (CTCF), 11581 (H3K27ac), 7 (H3K27me3), and 2 (P300), respectively (**Supplementary Figure 2F**). At the same time, we also showed the distribution of gain peaks and loss peaks in macrophages of the HBeAg-stimulated group (**Figures 6B–C**).

In addition, the results of KEGG analysis based on peak-associated genes showed that the differential peaks in the H3K27ac group was mainly enriched in pathways such as TNF signaling pathway and MAPK signaling pathway(**Figure 6D**), the CTCF group were mainly enriched in pathways such as Wnt signaling pathway and Endocytosis(**Supplementary Figure 3A**), the H3K27me3 group was mainly enriched in pathways such as Wnt signaling pathway and Hedgehog signaling pathway(**Supplementary Figure 3A**), and the P300 group was mainly enriched in pathways such as PPAR signaling pathway (**Supplementary Figure 3A**). Motif analysis of sequences underlying H3K27ac gain peaks revealed an enrichment for AP-1 and STAT-family transcription factors (**Figure 6E**), suggesting that HBeAg signaling culminates in STAT/AP-1 recruitment, subsequent H3K27 acetylation, and the anchoring of newly formed pro-inflammatory chromatin loops. In addition, the analysis of CTCF altered regions revealed a strong enrichment of binding motifs for the Forkhead box (FOX) family of transcription factors (e.g., FOXC2, FOXB1, FOXD1), as well as key immune regulators such as NFATC2 (**Supplementary Figure 3B**). This suggests that CTCF may cooperate with these specific co-factors to orchestrate 3D chromatin rewiring during macrophage activation. Conversely, motif analysis of the repressive H3K27me3 peaks exhibited a strikingly uniform and highly significant (Adjusted p = 0.000106) enrichment of the Helicase-like transcription factor (HLTF) motif, which was present in over 85% of the target sequences (**Supplementary Figure 3B**). This profound enrichment implicates a potential role for HLTF-associated chromatin remodeling in directing targeted Polycomb-mediated gene silencing (H3K27me3 deposition) in response to HBeAg stimulation. Together, these findings demonstrate that HBeAg orchestrates targeted epigenetic reprogramming, driving active enhancer deposition to reshape 3D chromatin architecture and macrophage phenotypes.

## 4 Discussion

HBV infection causes persistent liver damage through the chronic activation of the innate immune system, ultimately driving cirrhosis and hepatocellular carcinoma (HCC). Among HBV-derived proteins, our previous foundational studies established that HBeAg-rather than HBsAg or HBcAg-acts as the primary driver of macrophage activation and specific inflammatory cytokine release (3–5). However, the nuclear mechanisms by which HBeAg orchestrates this prolonged macrophage activation have remained obscure. In this study, we integrated whole-transcriptome sequencing, Hi-C, and ChIP-seq to provide the first comprehensive multi-omics map of Hepatitis B e Antigen (HBeAg)-stimulated macrophages. Our findings reveal that HBeAg drives a profound inflammatory and immune-tolerant phenotype not merely through simple signal transduction, but through a highly coordinated, hierarchical reorganization of the 3D genome, tightly coupled with targeted epigenetic remodeling.

### 4.1 HBeAg induces a pro-inflammatory microenvironment for immune escape

Transcriptome analysis confirmed that HBeAg induces a robust pro-inflammatory signature, heavily featuring the TNF, TLR, and specific cytokine-receptor signaling pathways. While acute inflammation is essential for viral clearance, persistent TNF signaling in the context of chronic HBV infection represents a double-edged sword. As highlighted in our gene network analysis, TNF acts as a central dysregulated node. Chronic TNF exposure is known to promote immune tolerance by exacerbating T cell exhaustion and recruiting regulatory T cells (15). Therefore, our transcriptomic data support the hypothesis that HBeAg manipulates macrophages to produce a specific inflammatory milieu, paradoxically facilitating HBV immune escape (16).

### 4.2 Macro-scale chromatin reorganization drives disease-associated gene expression

Crucially, our Hi-C data demonstrate that this transcriptomic rewiring is physically underpinned by global 3D chromatin reorganization. At the macro-scale, we observed extensive A/B compartment switching and dynamic TAD restructuring. The transition from the inactive (B) to the active (A) compartment is highly correlated with the *de novo* accumulation of H3K27ac (an active enhancer mark). This structural-epigenetic synergy directly drove the upregulation of key functional genes (26), such as MET and FHOD3. MET (c-Met) is a well-known receptor tyrosine kinase involved in macrophage polarization, motility, and inflammatory responses (27, 28). Concurrently, the restructuring of TADs—particularly the formation of “merged TADs”—exposed genes like FLNB and SESN2 to novel enhancer elements, leading to their robust upregulation. SESN2 (Sestrin 2) is a stress-inducible metabolic regulator (29), while FLNB is critical for cytoskeletal remodeling (30). FLNB can participate in the repair of vascular damage by interacting with glycoprotein Ib alpha (30). At the same time, mutations in this gene are closely related to Larsen syndrome and bone defects and bone development (31, 32). In addition, in terms of liver disease, studies have shown that FLNB expression is negatively correlated with the survival of patients with hepatocellular carcinoma (33). In studies of alcohol-induced hepatocellular carcinoma, ethanol can regulate the expression of FLNB in hepatocytes, thereby affecting the development of hepatocellular carcinoma (34). Importantly, the upregulation of these core genes was validated in human THP-1 macrophage datasets (GSE179618, GSE83148), underscoring an evolutionarily conserved spatial epigenetic mechanism utilized by HBV to reprogram host cell functions.

### 4.3 Chromatin loop rewiring dismantles antiviral and metabolic defenses

Beyond large-scale domains, fine-scale enhancer-promoter communication was also significantly altered via the rewiring of chromatin loops. Interestingly, while HBeAg stimulation formed new loops to activate inflammatory pathways, it simultaneously disrupted pre-existing intrachromosomal loops, leading to the targeted downregulation of specific genes, such as KBTBD11 and BLVRB. BLVRB (Biliverdin reductase B) is intrinsically linked to cellular redox homeostasis (35). Consistent with our research, previous studies have also demonstrated that BLRRB expression is upregulated in HBV-related liver fibrosis (36).The loop-mediated disruption of BLVRB expression—which was consistently observed in human HBV patient samples—suggests that HBeAg might impair the macrophage’s antiviral oxidative burst by dismantling the spatial architecture of protective genes. Thus, the 3D chromatin rewiring acts as a precise bi-directional switch: structurally assembling inflammatory hubs while physically disassembling specific antiviral defense nodes. Previously associated KBTBD11 with adipogenesis (37), its suppression in our model provides a spatial explanation for macrophage metabolic dysregulation during chronic HBV. We hypothesize that by epigenetically unlooping and silencing metabolic regulators like KBTBD11, HBeAg forces a metabolic shift in macrophages (e.g., altered lipid droplet formation or glycolysis), which not only fuels continuous cytokine production but also creates an immunosuppressive, lipid-rich microenvironment conducive to liver diseases.

### 4.4 AP-1 and STAT drive the signaling-to-epigenetic cascade

Finally, our multi-omic integration sheds light on the underlying molecular drivers of this 3D spatial reorganization. The deposition of H3K27ac at newly activated regions was highly enriched for AP-1 and STAT motifs, indicating that HBeAg signaling culminates in the recruitment of these specific transcription factors to anchor newly formed pro-inflammatory chromatin loops. Based on these findings, we propose a specific, testable signaling-to-epigenetic cascade: HBeAg initially triggers macrophage surface receptors (likely via TLRs), activating downstream MAPK and JAK/STAT kinase cascades. This results in the nuclear translocation of AP-1 and STAT, which recruit histone acetyltransferases (such as p300) to license latent enhancers via H3K27 acetylation. These newly activated enhancers subsequently recruit CTCF to pioneer *de novo* chromatin loops, physically bridging distant inflammatory promoters (such as those governing the TNF and Wnt signaling pathways) to drive the cytokine storms characteristic of HBV-related liver damage. Future studies utilizing specific MAPK/STAT chemical inhibitors prior to Hi-C mapping are required to validate this spatial-transcriptional axis.

### 4.5 Limitations and future directions

While this multi-omics snapshot offers novel insights, several strict limitations must be acknowledged. First, to mitigate the biological variance inherent in the limited sample size (n=2) of our primary murine RNA-seq, we utilized robust dispersion estimators in the DESeq2 algorithm. To rigorously compensate for this limitation, we did not rely on RNA-seq alone; all key findings (Met, Flnb, Kbtbd11) were multi-omics cross-validated using Hi-C structural shifts, ChIP-seq enhancer marks, and external human datasets. Second, the immortalized murine RAW264.7 model, while providing the highly stable and reproducible baseline required for complex Hi-C mapping, cannot fully recapitulate primary human hepatic macrophage architecture. Furthermore, integrating mouse and human transcriptomic data introduces inherent species biases. To address this, we did not merge datasets for joint clustering; rather, human datasets were strictly used as an independent biological filter to identify only evolutionarily conserved 3D genome-driven changes.

Finally, this study establishes robust correlations between 3D conformation and transcription but lacks direct functional disruption. Future investigations must move beyond observational omics; employing targeted chromatin interaction disruption—such as engineered zinc fingers or CRISPR-Cas3 loop deletions—is necessary to definitively confirm whether the HBeAg-induced TAD merges and A/B compartment flips are the absolute causative factors of macrophage inflammatory dysfunction.

## 6 Conclusion

In summary, by mapping the 3D interactome, we transition from identifying *what* genes are altered by HBeAg to understanding *how* they are spatially reprogrammed. HBeAg acts as an architectural modifier of the macrophage genome, opening inflammatory compartments and merging structural TADs via enhancer licensing. These evolutionarily conserved epigenetic alterations offer a new spatial framework for understanding HBV pathogenesis and identify specific chromatin loops as latent therapeutic targets for ameliorating HBV-related liver disease.

## Data Availability

The datasets presented in this study can be found in online repositories. The names of the repository/repositories and accession number(s) can be found below: NCBI GEO repository (https://www.ncbi.nlm.nih.gov/geo/), accession numbers GSE179618 and GSE83148. The raw data supporting the conclusions of this article will be made available by the authors.
