# Supplementary material for: High-resolution mapping of chromatin conformation in HBeAg-treated macrophage provides insights into pathogenesis of HBV-related liver diseases
Source: Front Immunol. 2026 Jul 16;17:1871642. doi: 10.3389/fimmu.2026.1871642 (PMC13422226; doi:10.3389/fimmu.2026.1871642)
Supplement: Supplementary file 1 [file DataSheet1.pdf]

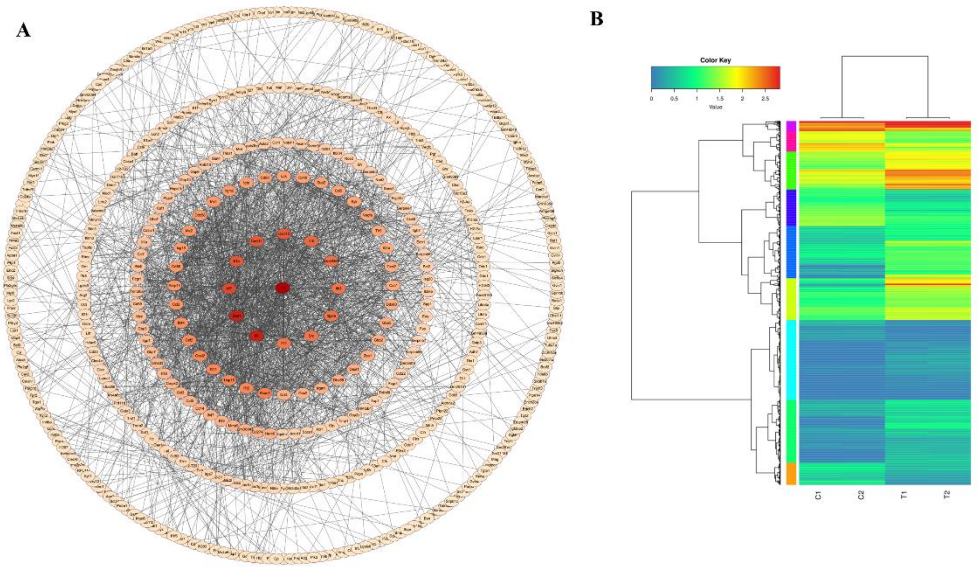

Figure S1. Supplementary detailed clustering and interaction networks of transcriptionally altered genes.

(A) Cluster diagram of gene expression patterns of differentially expressed genes. (B)Differentially expressed gene interaction network.

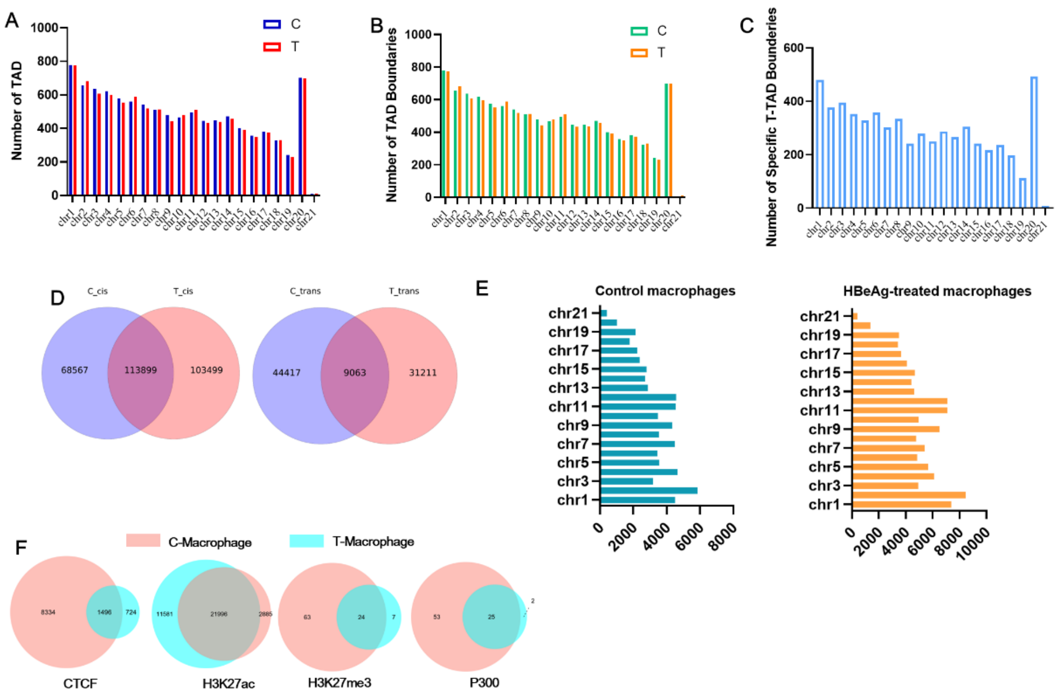

Figure S2. Genome-wide statistics of TADs, chromatin loops, and epigenetic peaks.

(A) The number of TADs on each chromosome in control and HBeAg-treated macrophages. (B) The number of TAD boundaries on each chromosome in control and HBeAg-treated macrophages. © The number of specific TAD boundaries on each chromosome in HBeAg-treated macrophages. (D) Venn diagram of intrachromosomal and interchromosomal chromatin loops in control macrophages and HBeAg-treated macrophages. (E) The number of intrachromosomal specific loops

on each chromosome in control macrophages and HBeAg-treated macrophages. (F) Venn diagram of the number of peaks in CTCF, H3K27ac, H3K27me3 and P300 in the two groups of samples.

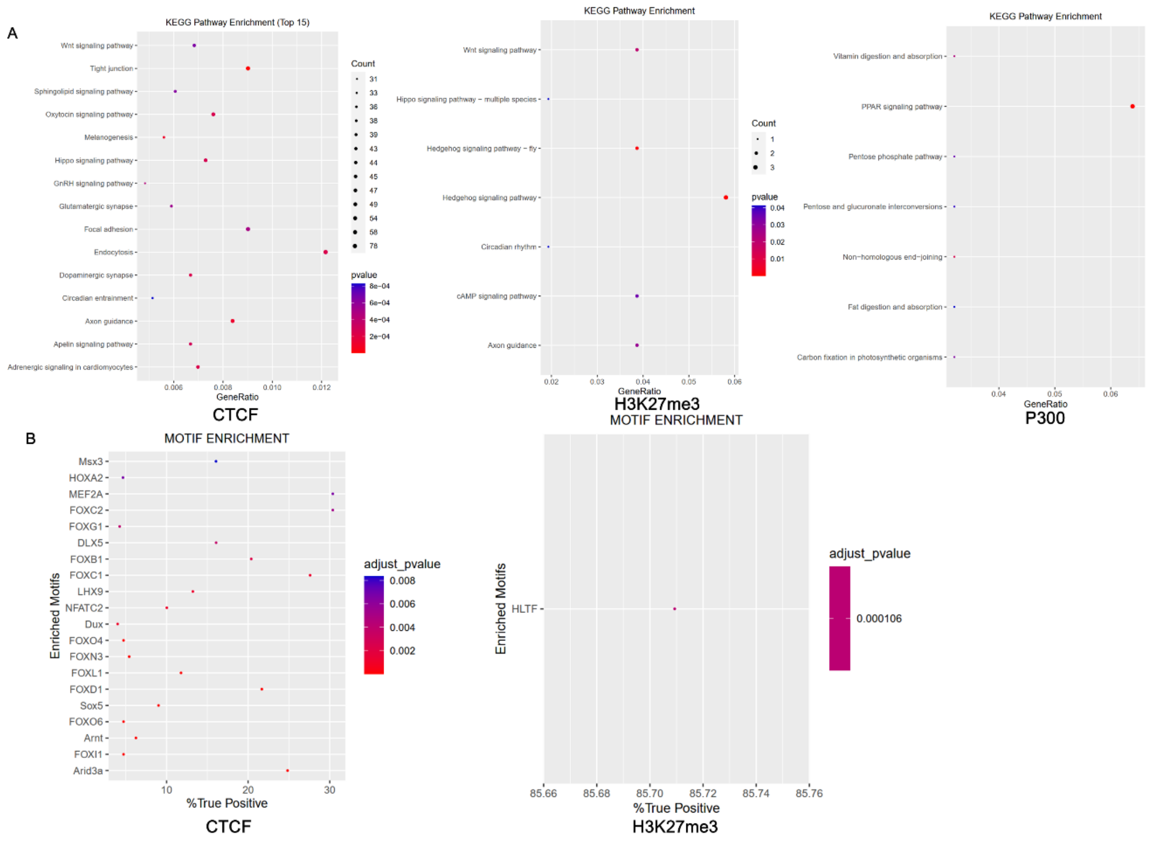

Figure S3. Supplementary pathway and motif enrichment analysis of specific epigenetic peaks. (A) KEGG analysis of enriched pathways of genes associated with differential peaks in CTCF, H3K27me3 and P300. (B) Motif enrichment results related to gain peaks in CTCF and H3K27me3.
